# Special Issue: Cancer Biomarkers and Targets in Digestive Organs

**DOI:** 10.3390/biomedicines7010003

**Published:** 2019-01-02

**Authors:** Nelson S. Yee, Nikki P. Lee

**Affiliations:** 1 Division of Hematology-Oncology, Department of Medicine, Penn State Health Milton S. Hershey Medical Center, Experimental Therapeutics Program, Penn State Cancer Institute, The Pennsylvania State University College of Medicine, Hershey, PA 17033, USA; 2 Department of Surgery, The University of Hong Kong, Hong Kong, China; nikkilee@hku.hk

## 1. Introduction

The identification and development of cancer biomarkers and targets have greatly accelerated progress towards precision medicine in oncology. Studies of tumor biology have not only provided insights into the mechanisms underlying carcinogenesis, but have also led to discovery of molecules that have been developed into cancer biomarkers and targets. Multi-platforms for molecular characterization of tumors and blood-based biopsies have greatly expanded the portfolio of potential biomarkers and targets. These cancer biomarkers have been developed for diagnosis, early detection, prognosis, and prediction of treatment response. The molecular targets have been exploited for anti-cancer therapy with proven benefits in improving treatment response and survival. However, plenty of research opportunity exists for discovering, developing, and validating cancer biomarkers and targets for improving the clinical outcomes of patients with malignant diseases, particularly those in the digestive system.

## 2. Cancer Biomarkers and Targets in Digestive System

Pancreatic-hepato-biliary and gastrointestinal carcinoma are among the most lethal human malignant diseases [1]. With the advance in developing tumor biomarkers and targets, progress has been made to improve treatment response and survival for patients with cancer of the digestive system [2,3,4,5,6,7]. In clinical practice, a few biomarkers and targets have been utilized for patients with cancers of digestive organs. Serum levels of carcinoembryonic antigen (CEA), carbohydrate antigen 19-9 (CA 19-9), and alpha-fetoprotein (AFP) have been clinically used as tumor markers of gastrointestinal and hepato-pancreatic-biliary malignancies [8,9,10]. Yet the sensitivity and specificity of these biomarkers for disease diagnosis and prognosis are somewhat limited. However, there are several clinically developed predictive biomarkers of treatment response. For instance, the cell-surface human epidermal growth factor receptor 2 (HER2) when amplified or over-expressed, has been targeted for treatment using the anti-HER2 antibody, trastuzumab, with proven survival benefit in gastric carcinoma [11]. Expression of programmed death-ligand 1 (PD-L1) in gastric carcinoma predicts therapeutic responsiveness of the anti-PD-1 antibody, pembrolizumab [12]. Wild-type K-RAS in colorectal carcinoma predicts clinical benefits of the anti-epidermal growth factor receptor antibodies, cetuximab [13] or panitumumab [14]. Deficiency in mismatch repair protein, or a high level of microsatellite instability in colorectal carcinoma, suggest treatment response using anti-PD-1 antibody, pembrolizumab [15], or nivolumab [16]. In recent years, studies have been conducted to explore and develop molecular biomarkers and targets in gastrointestinal cancers. Intense research for clinical translation is ongoing, with the goal of attaining the goal of precision care for patients with cancers in digestive organs.

## 3. Recent Advances in Gastrointestinal Oncology

This Special Issue of *Biomedicines* comprises a variety of articles about recent advances in the discovery, characterization, translation, and clinical application of cancer biomarkers and targets in the digestive system. These articles include original research, reviews, case studies, and conference papers. At the Multi-Disciplinary Patient Care in Gastrointestinal Oncology conference in Hershey, Pennsylvania, the new frontiers in various aspects of digestive organ cancers were presented [17]. In this meeting report, Yee et al. provide updates and discuss advances in the epidemiology and genetics, diagnostic and screening evaluation, treatment modalities, and supportive care for patients with gastrointestinal cancers. In a critical review, Zhang et al. present new perspectives of the development of biomarkers for gastrointestinal cancers [18]. The biomarkers, including those derived from tumor genome, tumor-associated microenvironment, and liquid biopsies, are discussed. Complementary to the review on biomarkers, Yee presents an up-to-date report of the systemic treatment of gastrointestinal malignancies [19]. In this conference paper, results and implications of the recent clinical trials that investigated the efficacy of chemotherapy, targeted therapeutics, and immunotherapy in pancreatic, gastroesophageal, biliary tract, hepatocellular, and colorectal carcinoma are discussed. In addition to this, Tchelebi et al. provide an overview of the role of stereotactic body radiation therapy (SBRT) in the management of malignant diseases in the upper gastrointestinal tract [20]. Moreover, the emerging data on biomarkers of immunotherapy and SBRT are evaluated, with a focus on pancreatic and hepatocellular carcinoma.

## 4. Biomarkers and Targets in Cancer of Digestive Organs

A number of articles in this Special Issue examine the biomarkers and targets with a focus on cancer in individual organs, including liver. While liver transplantation is a potentially curative treatment of hepatocellular carcinoma, liver graft injury has been identified as an acute phase event that leads to post-transplant tumor recurrence. Lee et al. examined this acute phase event at the molecular level by transcriptomic analysis of liver grafts from recipients with or without tumor recurrence following liver transplantation [21]. This study reveals the altered genetic expression in liver grafts, and paves the way to identify key molecular pathways that may be involved in post-transplant tumor recurrence. On the other hand, Posadas et al. demonstrate the potential value of tumor molecular profiling for individualized therapy in hepatocellular carcinoma [22]. In this patient case study, the treatment response as determined by progression-free survival appears to correlate with the differential expression of biochemical markers and genetic mutations of the tumors.

Besides hepatocellular carcinoma, several articles focus on cancer biomarkers and targets in the gastrointestinal tract. Fonkoua and Yee present a critical review of the molecular characterization of gastric carcinoma by the Cancer Genome Atlas Research Network, the Asian Cancer Research Group, and tumor molecular profiling through expression analysis and genomic sequencing of tumor DNA [23]. These molecular analyses have generated a number of potential biomarkers and targets that may be translated into clinical use. Moreover, patient cases of gastroesophageal carcinoma are reported to demonstrate survival advantage of molecular profile-based treatment, suggesting the potential value of tumor molecular profiling in guiding selection of therapy tailored to the individual patient. For colorectal carcinoma, Zhang et al. evaluate circulating tumor cells and their expressed genes as biomarkers, along with assessment of the clinical outcomes [24]. Results of this study show that circulating tumor cells and their expression of both endothelial and tumor progenitor cell biomarkers are potential prognostic biomarkers in colorectal cancer. Complementary to clinical investigation in humans, Lu et al. described the zebrafish model to study human intestinal disorders and tumors [25]. In this review article, mutant and transgenic zebrafish as well as xenograft models as an in vivo platform for understanding the pathogenesis of gastrointestinal diseases and for evaluation of anti-cancer drugs are discussed.

Despite advances in developing clinically useful biomarkers and targets in gastrointestinal cancers, relatively little progress has been made for patients with pancreatic carcinoma. While early detection of pancreatic carcinoma is critical for improving patient survival, agents that selectively target pancreatic tumor are expected to enhance therapeutic efficacy. In this Special Issue, Matters and Harms present a detailed review of G protein-coupled receptors, which are key target proteins for drug discovery. They further discuss the potential of GPCRs as biomarkers for tumor imaging and targeted treatment of pancreatic carcinoma [26].

## 5. Conclusions and Future Perspectives

Research on discovery and development of cancer biomarkers and targets has been steadily progressing. Rigorous investigation for identification and validation of biomarkers and targets in both preclinical models and clinical studies are expected to generate new opportunities for making a positive impact on survival and quality of life in the patients. The articles in this Special Issue provide an update on the frontiers in gastrointestinal oncology, with a focus on biomarkers and targets in cancers of the digestive system. We hope this Special Issue will help stimulate research collaboration on developing strategies for prevention, early detection, diagnosis, and screening of cancers in digestive organs, as well as improving treatment outcomes and psychosocial support in patients with these malignant diseases. In particular, liquid biopsy for cancer biomarkers and targets has been a major focus of research with translation into clinical applications.

Recent advances in plasma-derived extracellular vesicles (EVs) have demonstrated the potential of making a clinically meaningful impact in the field of cancer biomarkers and targets. Analysis of EV-derived molecular markers is complementary to the conventional diagnostic modalities. By application of nano-, micro-, digital-, and microarray-based technologies, multiplex analysis of disease-specific markers is expected to improve the sensitivity and specificity of bodily fluid-based biopsies for diagnosis of cancer. These minimally invasive diagnostic tools that utilize ultra-low sample volume may prove to be economically cost effective for screening of cancer in the high-risk population and even in the general population. In addition to this, increasing evidence has indicated the potential value of blood-based biopsies in combination with tumor molecular profiling for developing predictive biomarkers of treatment response, as well as personalized targets of therapy. Further development, optimization, and clinical validation of these cancer biomarkers and targets will hopefully enable us to attain the goal of precision medicine in cancer of digestive organs.
